# The genome of Mekong tiger perch (*Datnioides undecimradiatus*) provides insights into the phylogenetic position of Lobotiformes and biological conservation

**DOI:** 10.1038/s41598-020-64398-2

**Published:** 2020-05-18

**Authors:** Shuai Sun, Yue Wang, Wenhong Zeng, Xiao Du, Lei Li, Xiaoning Hong, Xiaoyun Huang, He Zhang, Mengqi Zhang, Guangyi Fan, Xin Liu, Shanshan Liu

**Affiliations:** 1 BGI-Qingdao, BGI-Shenzhen, Qingdao, 266555 China; 2 BGI-Shenzhen, Shenzhen, 518083 China; 3 China National GeneBank, BGI-Shenzhen, Shenzhen, 518120 China; 4 Jiangxi University of Traditional Chinese Medicin, Nanchang, 330004 China; 5 School of Future Technology, University of Chinese Academy of Sciences, Beijing, 101408 China; 6 BGI Education Center, University of Chinese Academy of Sciences, Shenzhen, 236009 China

**Keywords:** DNA sequencing, Phylogenetics, Conservation biology

## Abstract

Mekong tiger perch (*Datnioides undecimradiatus*) is an ornamental and vulnerable freshwater fish native to the Mekong basin in Indochina, belonging to the order Lobotiformes. Here, we generated 121X stLFR co-barcode clean reads and 18X Oxford Nanopore MinION reads and obtained a 595 Mb Mekong tiger perch genome, which is the first whole genome sequence in the order Lobotiformes. Based on this genome, the phylogenetic tree analysis suggested that Lobotiformes is more closely related to Sciaenidae than to Tetraodontiformes, resolving a long-time dispute. We depicted the genes involved in pigment development in Mekong tiger perch and results confirmed that the four rate-limiting genes of pigment synthesis had been retained after fish-specific genome duplication. We also estimated the demographic history of Mekong tiger perch, which showed that the effective population size suffered a continuous reduction possibly related to the contraction of immune-related genes. Our study provided a reference genome resource for the Lobotiformes, as well as insights into the phylogenetic position of Lobotiformes and biological conservation.

## Introduction

Mekong tiger perch (*Datnioides undecimradiatus*) is one tropical freshwater fish, belonging to the order Lobotiformes under series Eupercaria^1^. It is native to the Mekong river and usually found in the main waterway and large tributaries of the Mekong river basins, feeding on small fishes and shrimps^2^. It is also one of ornamental fish, which is featured for its vertical white-yellow or green and black stripes running its body.

Eupercaria is by far the largest series of percomorphs with more than 6,600 species arranged in 161 families and at least 16 orders. The phylogenetic relationship of the order Lobotiformes, Tetraodontiformes, and the family Sciaenidae is in dispute. One previous study suggested that Sciaedidae was the sister clade of Tetraodontiformes and then followed by Lobotiformes based on 44 DNA makers from uncompleted nuclear and mitochondrial sequences combined with morphological characters^3^. Compared to it, Lobotiformes was reported to be more closely related to Tetraodontiformes than to Sciaedidae using molecular and genomic data, which were also not whole-genome sequences for most of the species^4^. However, more recently Lobotiformes was reported to be more closely related to Sciaenidae than to Tetraodontiformes based on complete mitochondrial genome and transcriptomic data^5,6^. Apart from that, fourteen families of Eupercaria included in order-level *incertae sedis*, which are called “new bush at the top”, were not assigned to explicit phylogenetic position^7^. Furthermore, reliable delimitation of order and family, and phylogenetic polytomy were a long-term issue^8^. Therefore, the whole-genome sequences containing comprehensive evolutionary information are called for resolving the long-time dispute on the phylogeny of the huge number of species in Eupercaria, especially for the problem of “new bush at the top”.

In addition to its utility for resolving the evolutionary history of Eupercaria, Mekong tiger perch has a skin color pattern with vertical white-yellow or green and black stripes running its body. Skin color diversity in animals has important functions in numerous biological processes and social behaviors, such as sexual selection, kin recognition and changing coloration for camouflage^9^. Recent studies proposed that teleost genomes might contain more copies of genes involved in pigment cell development than tetrapod genomes after an ancient fish-specific genome duplication (FSGD), which might contribute to the evolution and diversification of the pigmentation gene repertoire in teleost fish^10^. With more genome sequences, especially for fish with unique skin color schemes such as Mekong tiger perch, comparative genomics could be further applied to illustrate the genetic mechanisms of skin color development.

Mekong tiger perch is currently assigned as ‘Vulnerable (VN)’ on INCN red list due to the rapidly declined population size^11^, and is considered as ‘endangered (EN)’ on Thailand Red Data^2^. The external factors, such as the construction of hydraulic engineering infrastructures, urban pollution, and the aquarium trade, are thought to be exerting a negative effect on wild populations. Meanwhile, internal genetic factors such as resistance to biological and abiotic stress play a role in their survival. Due to its limited distribution and commercial values, rare genetic research was focused on Mekong tiger perch. With the rapid development of genomics, each fish deserves the right to own its sequenced genome representing its unique genetic resource, which can be applied to better investigate its unique characters and biological conservations.

Here, we sequenced Mekong tiger perch and assembled a reference genome, which was the first genome of the order Lobotiformes. We constructed a phylogenetic tree in Eupercaria based on the whole genome sequences to elucidate the relationships among family Sciaenidae, order Lobotiformes and order Tetraodontiformes, providing insights into the phylogenetic position of Lobotiformes. Utilizing the assembled genome, we identified genes involved in pigment development in Mekong tiger perch. We also confirmed the continuous reduction of population size by analyzing the demographic history and found the contraction of immune-related genes might be a contributing factor for Mekong tiger perch’s vulnerability. The genome assembly of Mekong tiger perch provided a valuable genome resource for further fish studies in Lobotiformes, and also contributes to the understanding of skin color development as well as demographic history and biological conservation.

## Results

### Genome assembly, annotation, and genomic features

**Figure 1:**
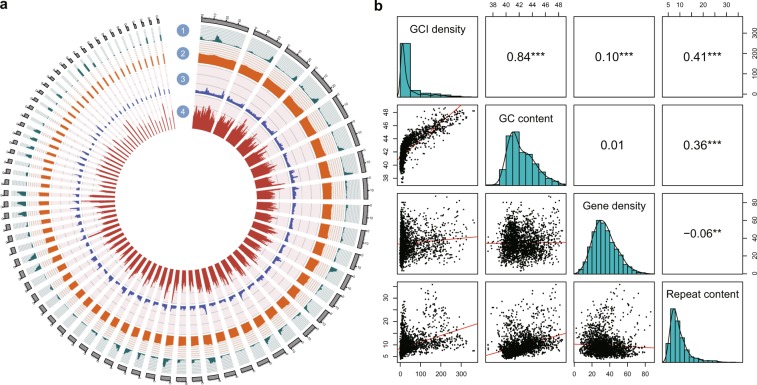
The genome features of *D. undecimradiatus*. **(a)** A Circos plot representing four features using sliding overlapping windows of 1 Mb length with 200 kb step through the 72 scaffolds (scales in Mb), which accounts for more than 90% of genome length. (1) CGI content, measured by CGIs number per million base pairs (megabase, Mb). The range of the axis is 0 to 500. (2) GC content, measured by the proportion of GC in unambiguous bases of 1 Mb window size. The range of the axis is 0 to 100. (3) Repeat content, measured by the proportion of repeat regions of 1 Mb window size. The range of the axis is 0 to 100. (4) Gene density, measured by genes number per million base pairs. **(b)** Correlation matrix plot with significance levels between four genome features. The lower triangular matrix is composed by the bivariate scatter plots with a fitted linear model. The diagonal shows the distribution by histogram with density curve. The upper triangular matrix shows the Pearson correlation plus significance level. Different significance levels are highlighted with asterisks: *p*-values 0.001 (***), 0.01 (**), 0.05 (*). This plot was generated with the “psych” package in R (v3.5.0).

We sampled muscle tissue from a Mekong tiger perch captured in the Mekong river (Supplementary Fig. S1) and applied single tube long fragment read (stLFR)^12^ technology for whole genome sequencing, generating 122.4 Gb stLFR co-barcode raw reads. After filtering low-quality and duplicated reads, we obtained 75.3 Gb clean data (121X depth) for genome assembly using supernova^13^ and closed gaps using GapCloser^14^. We also generated 11 Gb Oxford Nanopore MinION reads (18X depth) to further fill the gaps using TGSGapFiller^15^. A final genome assembly spanning 595 Mb was obtained, accounting for 95.5% of the estimated genome size (623 Mb, Supplementary Fig. S2). The assembly achieved a high level of contiguity, with a total of 4,442 scaffolds and scaffold N50 of 9.69 Mb. The longest 72 scaffolds (longer than 1.38 Mb) accounted for 90% of the total genome, and the longest scaffold reached up to 39.22 Mb (Fig. 1a, Table 1, Supplementary Table S1). Total repeat content accounted for 10.10% of the genome, and 21,160 protein-coding genes were predicted via *ab initio* and homology-based methods (Table 2, Supplementary Table S2). The average length of coding sequences (CDS) was 1,846 bp with an average of 10.88 exons per gene, which were similar to that of other related species (Supplementary Fig. S3, Supplementary Table S3). The ncRNAs including miRNA, tRNA, rRNA, and snRNA were also annotated with a total length of 194.65 kb (Supplementary Table S4). We used BUSCO metazoan database (v9) to evaluate the completeness of gene set and observed completeness of 95.19%. Furthermore, the mitochondrial genome was assembled with a total length of 16,606 bp, containing 18 coding genes, 2 rRNA, and 17 tRNA (Supplementary Table S5).

**Table 1:** Assembly of Mekong tiger perch genome.

	stLFR		stLFR + nanopore	
	contig	scaffold	contig	scaffold
Number	10,550	4,442	5,009	4,442
Length (bp)	582,060,642	594,964,832	593,074,541	595,051,252
Maximum length (bp)	1,134,184	39,306,008	13,208,434	39,224,198
Average length (bp)	55,172	133,941	118,402	133,960
N50	995	18	86	18
NL50	171,232	9,730,178	2,175,996	9,689,676
N90	3,706	71	302	72
NL90	37,538	1,407,639	447,174	1,380,814
N rate (%)	0.00	2.17	0.00	0.33
GC content (%)	42.74	42.74	42.77	42.77

**Table 2:** Repeat and gene annotation of Mekong tiger perch genome.

Repeat content		Genes prediction and annotation	
DNA (bp / %)	36,324,964 / 6.11	Gene number	21,160
LINE (bp / %)	16,349,136 / 2.75	Average gene length (bp)	14,391
SINE (bp / %)	1,152,466 / 0.19	Average mRNA length (bp)	1,846
LTR (bp / %)	14,084,966 / 2.37	Average exon number per gene	11
Other (bp / %)	4,319 / 0.00	Average exon length (bp)	170
Unknown (bp / %)	19,939,915 / 3.35	Average intron length (bp)	1,2691,523
Total (bp / %)	71,231,464 / 11.97	Function annotated genes	19,853

CpG islands (CGIs) are an important group of CpG dinucleotides in the guanine and cytosine rich regions as they harbor functionally relevant epigenetic loci for whole genome studies. 32,148 CpG islands (CGIs) were identified with a total length up to 18.8 Mb. The CpG density had the most prominent correlations with three other genomic features. It positively correlated with GC content, gene density, and repeat content (Fig. 1b, Supplementary Table S6), showing a similar pattern observed in other published fish and mammals^16–18^.

### Eupercaria phylogenetic tree, genome-wide synteny, and gene trees uncover the phylogenetic position of Lobotiformes

**Figure 2:**
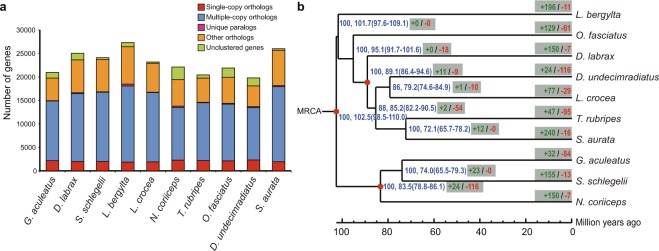
Comparative genomics and phylogeny analysis for *D. undecimradiatus* and other related species. **(a)** Gene number distributed in five types of gene families. Single-copy orthologs include the common orthologs with one copy in the nine species, multiple-copy orthologs include the common orthologs with different copies in the nine species, unique paralogs include the genes families only existed in one species, other orthologs include the orthologs with different copies in more than two but not all species, and unclustered genes include the genes that cannot be assigned into gene families. **(b)** Time-calibrated maximum likelihood phylogenetic tree of nine species from seven orders in Eupercaria. Red nodes represent the calibration time points. The two numbers separated by comma on the right of the nodes represent bootstrap values and estimated divergent times (mean and 95% highest-probability), respectively. Behind the divergent time, the green positive number and the red negative number in grey boxes stand for the number of significantly expanded and contracted gene families, respectively. Abbreviations: *O. fasciatus* (*Oplegnathus fasciatus*), *L. bergylta* (*Labrus bergylta*), *D. undecimradiatus* (*Datnioides undecimradiatus*), *D. labrax* (*Dicentrarchus labrax*), *G. aculeatus* (*Gasterosteus aculeatus*), *N. coriiceps* (*Notothenia coriiceps*), *S. schlegelii* (*Sebastes schlegelii*), *L. crocea* (*Larimichthys crocea*), *T. rubripes* (*Takifugu rubripes*), *S. aurata* (*Sparus aurata*).

To clarify the evolutionary relationships of major orders in Eupercaria, nine sequenced species from 8 different orders were used for comparative genomics analysis (Supplementary Table S7). We clustered gene families based on protein sequence similarity and obtained a total of 13,615 gene families, 1,291 of which were single-copy gene families (Fig. 2a, Supplementary Table S8). A total 893,287 sites from multi-alignment on the first phase codon of those single-copy gene families were used to construct the maximum likelihood (ML) tree. The phylogeny of the eight orders was found consistent with two previous studies^5,6^. Perciformes was identified as an early split branch compared to other orders in Eupercaria, and the divergent time was estimated 102.5 million years ago (mya) (Fig. 2b). Our phylogenetic tree showed Lobotiformes was more closely related to Sciaenidae than to Tetraodontiformes (Fig. 2b), supporting several previous studies^5,6^. Furthermore, the divergent time between Lobotiformes and Sciaenidae was inferred to be 79.2 mya (Fig. 2b).

**Figure 3:**
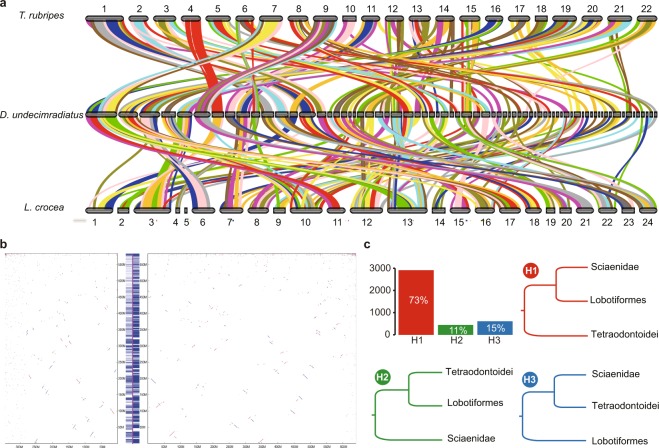
Syntenic analysis for *D. undecimradiatus* and other related species. **(a)** synteny of *D. undecimradiatus* between *L. crocea* and *T. rubripes* at whole-genome gene-level and different colors represent different synteny blocks. **(b)** Synteny of *D. undecimradiatus* between *L. crocea* and *T. rubripes* at whole-genome nucleotide-level. The y-axis represents the *D. undecimradiatus* genome, and the left x-axis refers to *T. rubripes* genome and right x-axis refers to *L. crocea* genome. The fringe plot on the left of y-axis represents the syntenic blocks between *D. undecimradiatus* and *T. rubripes* on *D. undecimradiatus* genome and the fringe plot on the right of y-axis represents the synteny regions between *D. undecimradiatus* and *L. crocea*. **(c)** The number and percentage of gene trees that support three hypotheses concerning the phylogenetic position of Lobotiformes.

The conserved genomic synteny could reflect the sequence arrangements on evolutionary processes and be used to further demonstrate the ambiguous phylogeny among Lobotiformes (*Datnioides undecimradiatus*) and closely related Sciaenidae (*Larimichthys crocea*) and Tetraodontiformes (*Takifugu rubripes*). The syntenic analysis on both whole-genome gene-level and nucleotide-level were performed by aligning *L. crocea* and *T. rubripes* to our assembled *D. undecimradiatus*, separately. On whole-genome gene-level, after remaining syntenic blocks with more than 3 genes, 96.20% of *D. undecimradiatus* genes showed synteny with *L. crocea* with an average of 40.79 genes per block, while only 91.60% of *D. undecimradiatus* genes had synteny with *T. rubripes* with an average of 35.11 genes per block (Fig. 3a, Supplementary Table S9). Similarly, on whole-genome nucleotide-level, after filtering out syntenic blocks less than 1 kb, 41.76% of *D. undecimradiatus* genome sequences were covered by *L. crocea* genome with an average of 2.29 kb per block. In comparison, only 10.48% of *D. undecimradiatus* genome sequences were covered by *T. rubripes* genome with an average of 1.65 kb per block (Fig. 3b, Supplementary Table S10). In addition, the distributions of the length of syntenic blocks at both whole-genome nucleotide-level and gene-level showed significant differences by t-test statistics (nucleotide-level, *p*-value<0.0001(***); gene-level, *p*-value < 0.05(*)) (Supplementary Fig. S4). Despite the difference in genome size, both *L. crocea* and *T. rubripes* genomes were assembled to comparable chromosomal level and the BUSCO assessments showed no significant differences in the completeness of genome and gene set between *L. crocea* and *T. rubripes* (Supplementary Table S11). Therefore, the results of synteny suggested that Sciaenidae had better evolutionary conservation and closer relationship with Lobotiformes compared with Tetraodontiformes, providing strong evidence for the constructed phylogenic tree (Fig. 2b).

The gene trees were also used to demonstrate the phylogeny among Lobotiformes, Sciaenidae, and Tetraodontiformes. Based on the above synteny results, only 3,974 homologous genes of 1:1:1 on the syntenic blocks were inferred as reliable orthologous genes to construct the gene trees, with human CDS as outgroup in the rooted tree. As a result, 73% of the 3,974 orthologous gene trees supported that *D. undecimradiatus* was more closely related to *L. crocea*, supporting the hypothesis that Lobotiformes was closer with Sciaenidae instead of the other two hypotheses (Fig. 3c).

### The genes involved in pigment development were identified and main rate-limiting genes of pigment synthesis retained two copies after FSGD similar to other teleosts

**Figure 4:**
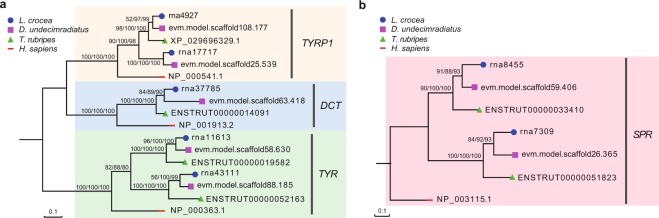
The phylogeny of four rate-limiting genes involved in pigment synthesis. **(a)** The concordant phylogenetic tree of tyrosinase family (*TYRP1*, *DCT*, and *TYR*) using maximum likelihood, neighbor-join and minimal evolutionary methods. The corresponding bootstrap values were showed on branch labels. **(b)** The concordant phylogenetic tree of *SPR* gene using maximum likelihood, neighbor-join and minimal evolutionary methods. The corresponding bootstrap values were showed on branch labels.

In consideration of special skin color pattern, among established pigmentation database containing 198 genes^19^, 172 genes were found in our genome, occupying 92% of the database and possibly establishing genetic resources to study the phenotypic characteristics of vertical white-yellow or green and black stripes running its body (Supplementary Table S12). Tyrosinase family (*TYR*, *DCT*, *TYRP1*) plays a role as rate-limiting genes in melanin synthesis pathway. Duplication of *TYR* and *TYRP1* was observed, and *DCT* is present as a single copy. (Fig. 4a). Meanwhile, for pteridine synthesis pathway, one main rate-limiting gene *SPR* also has two copies (Fig. 4b). Our findings suggest Mekong tiger perch retained some pigment-related genes after the fish-specific whole-genome duplication (FSGD), which showed similar gene retention patterns to other closely related teleosts^10^.

### Decreasing population size related to the contraction of immune-related gene families provides clues to biological conservation

**Figure 5:**
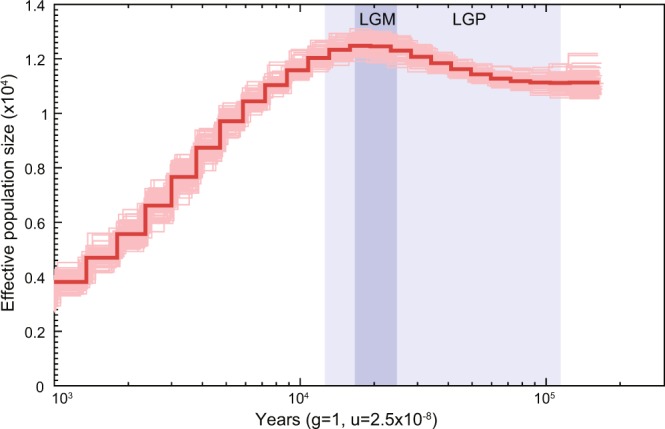
The demographic history of Mekong tiger perch inferred using PSMC. The LGM (last glacial maximum, ~26.5–19 kya) and LGP (last glacial period, ~115–11.7 kya) are shaded in purple and light purple respectively. Time scale on the x-axis is calculated assuming a mutation rate of 2.5 × 10^−8^ per generation and generation time equal to 1.

Pairwise sequentially Markovian coalescent (PSMC)^20^ was used to infer the demographic history of Mekong tiger perch. The effective population size continuously reduced since the last glacial maximum (LGM) and there were no signs of recovery to date (Fig. 5), which was consistent with its vulnerable state^2,11^.

**Figure 6:**
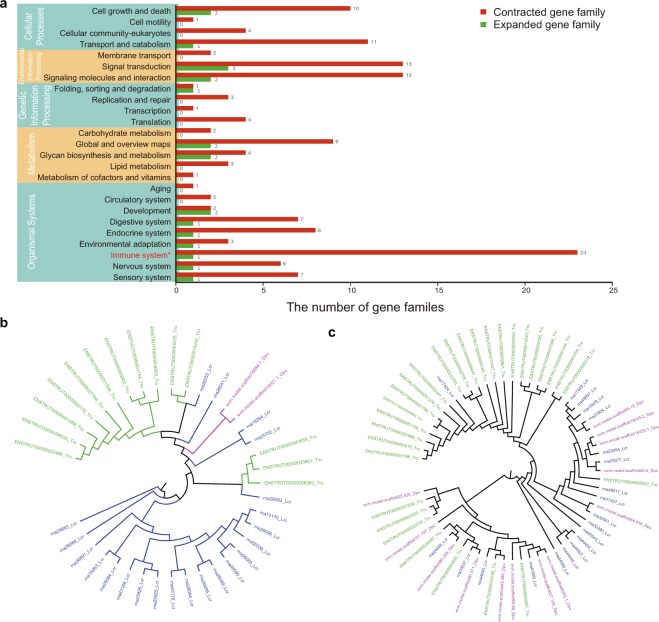
The genetic basis possibly related to the vulnerability of Mekong tiger perch. **(a)** The number of significantly contracted and expanded gene families (*p*-value <0.05 (*)) involved in different KEGG pathways (at level 1 and level 2). The number at the right end of the bar indicates the number of gene families. The most significantly expanded immune system pathway is shown in red font and asterisk. **(b)** Phylogenetic tree of *MHC I* gene family. Green color refers to *T. rubripes*, and blue and purple refers to *L. crocea* and *D. undecimradiatu* respectively. **(c)** Phylogenetic tree of *NLRP12* gene family. Green color represents *T. rubripes*, and blue and purple represent *L. crocea* and *D. undecimradiatu* respectively.

The change of gene copy number plays a role in species adaptation^21^. We identified the expanded and contracted gene families in Mekong tiger perch, and 19 and 101 significantly expanded and contracted gene families were found (*p*-value <0.05 (*)), respectively (Fig. 6a). 62 contracted and 18 expanded gene families were annotated with KEGG ortholog functions, among which 23 contracted gene families were involved in the immune-related pathway (Fig. 6b, Supplementary Tables S13 and S14). Furthermore, the immune-related gene families were annotated to *MHC I*, *NLRP12*, *ANK* (*ankyrin*), *IGH*, *CLDN*, and *PLAUR* (Supplementary Table S15 and S16), which may play a role in the adaptive immunity and survival. For example, *MCH1*, which is responsible for presenting peptides on the cell surface for T cells recognition^22^, was significantly contracted in *D. undecimradiatus* with only 2 copies, compared to 22 copies in closely related *L. crocea* and 14 copies in *T. rubripes* (Fig. 6b). *NLRP12*, which plays a role in regulating inflammation and immunity^23^, had 13 copies in *D. undecimradiatus*, compared to 20 copies in *L. crocea* and 29 copies in *T. rubripes* (Fig. 6c). The contraction of immune-related genes may affect the resilience of Mekong tiger perch to diseases or environmental stress, implying that species and habitat conservation for Mekong tiger perch is necessary.

## Discussion

The phylogeny of Eupercaria plays a fundamental role in species classification and uncovers the species evolutionary history at the Cretaceous–Palaeogene boundary^24^. However, although species in Eupercaria account for more than twenty percent of the bony fish, the Eupercaria phylogeny is ambiguous or conflicted, especially for the “new bush at the top”^4^. Meanwhile, the resolution of the phylogeny is currently limited to the order level, and few studies could go down to the class level or species level. Relying on limited morphological characters and molecular sequences, it is difficult to draw convincing conclusions^3–6^. In contrast, whole genome sequencing provides sufficient evolutionary information to perform the phylogenetic analysis of species. In our study, we clarified the relationship of Lobotiformes to its related family or order, Sciaenidae and Tetraodontiformes. With the rapid development of sequencing technology, large-scale genome sequencing projects are being initiated or achieved, such as Genome 10K^25^ and fish 10k project^26^, which will greatly promote the studies on fish classification and evolution.

Skin color is a biologically important trait, which is a fascinating research topic and has great implications on biological adaption, commercial value, and skin health^27,28^. In our study, most known genes involved in pigmentation development, regulation and synthesis can be found on our assembled genome. However, the research of underlying mechanisms is difficult to penetrate with very limited genome resources. More fish genome data and molecular experiments will facilitate the analysis of skin color regulation mechanisms.

Biological conservation is an important research field of the relationship between humans and nature^28^. Different species vary in their adaptive capacities. In our study, immune-related genes of Mekong tiger perch were significantly contracted compared to those in closely related species, indicating that the decreasing adaptive immunity in Mekong tiger perch may be potentially responsible for its vulnerability. Expansion of *MHC I* was widely identified as one compensatory immunity mechanism to the loss of *MHC II* in many fishes, such as Atlantic cod^29,30^. Furthermore, one previous study showed that a low copy number of *MHC I* have a low diversification rate^30^. In our study, the number of *MHC I* in Mekong tiger perch is greatly reduced to only 2 copies and it indicates that the level of species diversity of Mekong tiger perch may be at a lower level. Therefore, it is necessary for humans to take various measures to protect it, such as improving the living environment and artificial breeding, and thus help to maintain species diversity.

## Materials and Methods

### DNA extraction and stLFR library construction, and sequencing

The long genomic DNA molecules were extracted from the muscle of Mekong tiger perch using a conventional method for sufficient DNA quality^31^. The stLFR library was constructed following the standard protocol using MGIEasy stLFR library preparation kit (PN:1000005622)^12^. In detail, the transposons with hybridization sequences were inserted in the long DNA molecules approximately every 200–1000 base pairs. The transposon integrated DNAs were then mixed with beads that each contained an adapter sequence. A unique barcode was shared by all adapters on each bead with a PCR primer site and a capture sequence that was complementary to the sequences on the integrated transposons. When the genomic DNA was captured to the beads, the transposons were ligated to the barcode adapters. After a few additional library-processing steps, the co-barcoded sub-fragments were sequenced on the BGISEQ-500 sequencer. To generate long reads to overcome the gaps (long ambiguous sequences) induced by repeats, library preparation and sequencing were performed on the MinION nanopore sequencer (Oxford Nanopore Technologies, Oxford, UK) according to the base protocols from Oxford Nanopore.

The adult individual from the Mekong river was purchased from the farmers’ market in Lao People’s Democratic Republic on May 21, 2018, and sex was unknown. The experimental procedures were in accordance with the guidelines approved by the institutional review board on bioethics and biosafety of BGI (IRB-BGI). The experiment was authorized by IRB-BGI (under NO. FT17007), and the review procedures in IRB-BGI meet good clinical practice (GCP) principles.

### Reads filtering, genome size estimation, and genome assembly

We generated a total of 1,223,801,322 million raw pair-end co-barcoding reads of 122.4 Gb. To obtain a high-quality genome, SOAPnuke (v2.2)^32^ was performed to filter low-quality reads (>40% low-quality bases, Q < 7), PCR duplications, or adapter contaminations. After that, 753,357,182 clean pair-end reads remained. Based on the 17-mer analysis, the Mekong tiger perch genome size was estimated to be 623 Mb. Supernova assembler v2.0.1 (10X Genomics, Pleasanton, CA) was used to construct contigs and scaffolds, followed by gap closing using GapCloser (v1.2)^14^. We generated a total of 11.0 Gb long reads on the MinION nanopore sequencer and further filled the gaps using TGSGapFiller^15^ with default parameters.

### Repeats prediction, gene structure prediction, and gene function annotation

To predict repeat elements in the Mekong tiger perch genome, we used both *de novo* approaches and homology-based approaches. Firstly, we aligned our genome against the Repbase database^33^ at both protein and DNA levels by using RepeatMasker (v4.0.5) and RepeatProteinMasker (v4.0.5)^34^ to identify transcriptional elements (TEs). Secondly, we used RepeatModeler (v1.0.8)^35^ and LTR-FINDER (v1.0.6)^36^ to implement *de novo* repeat annotation. Next, we used RepeatMasker to complete repeat elements identification and classification. Lastly, we combined the above results.

We masked the repeats in Mekong tiger perch genome and gene prediction was performed using both homology-based and *ab initio* prediction. For homology-based annotation, we downloaded protein sequences of *Dicentrarchus labrax*, *Labrus bergylta*, *Larimichthys crocea*, and *Gasterosteus aculeatus* from NCBI. We aligned these sequence to Mekong tiger perch genome using BLAST^37^ with an E-value cutoff of 1e^−5^ and coverage >30% to identify homologous genes. Based on the aligned results, we used GeneWise (v2.4.1)^38^ to predict gene models. Furthermore, we used AUGUSTUS (v3.1)^39^ and GENSCAN (v2009)^40^ for *ab initio* prediction with default parameters and zebrafish data as a training set. Lastly, we integrated all above gene models by EVM^41^. We used BUSCO (v3.0.2)^42^ to assess gene annotation integrity using metazoan (v9) database.

To perform gene function annotation, we aligned the predicted gene sets against Kyoto Encyclopedia of Genes and Genome (KEGG, v87.0)^43^ and NR (v84)^44^ databases using BLASTP^37^ to identify genes with similar functions (E-value ≤ 1e-5). For identifying gene motifs and domains and obtaining Gene ontology (GO) terms^45^, we aligned our predicted genes against ProDom^46^, Pfam^47^, SMART^48^, PANTHER^49^, and PROSITE^50^ using InterProScan^51^.

### Prediction of ncRNA and CpG islands

Four types of ncRNA (Non-coding RNA), including tRNA, snRNA, miRNA, and rRNA were predicted. We used tRNAscan-SE (v1.3.1) to predict tRNA in our genome with default parameters. The genome was aligned against Rfam (v12.0) database (Nawrocki E P *et al*., 2015) and based on mapping results we used infernal (v1.1.1) (Nawrocki E P & Eddy S R, 2013) to infer snRNA and miRNA. We aligned vertebrate rRNA database against Mekong tiger perch genome to predict rRNA.

The CpG islands (CGIs), which are clusters of CpGs in CG-rich regions, were identified on genome wide using CpGIScan^52^ with the parameters “–length 500–gcc 55–oe 0.65”.

### Comparative genome analysis

We downloaded the annotation files of eight species including *Dicentrarchus labrax*, *Gasterosteus aculeatus*, *Labrus bergylta*, *Labrus bergylta*, *Notothenia coriiceps*, *Oplegnathus fasciatus*, *Larimichthys crocea*, *Takifugu rubripes*, and *Sparus aurata* form NCBI database (Supplementary Table S7). The longest transcript was extracted for each gene. We filtered out the sequences with length less than 50 amino acids, termination codon in the middle, and the sequence length not divisible by 3 to obtain high-quality gene sets for each species.

All-versus-all protein similarities were precomputed using BLASTP^37^ and TreeFam (v4.0)^53^ was used to identify gene families. We concatenated single-copy genes into a supergene matrix for all species and extracted sites on the first phase of codon to construct the phylogenetic tree using RAxML (v8.2.12)^54^ with GTRCATX nucleotide substitution model with parameters “-f a -x 12345 -p 12345 -# 500 -m GTRCATX”. With three species divergent time (splits between *Gasterosteus aculeatus* and *Larimichthys crocea*, *Dicentrarchus labrax* and *Larimichthys crocea*, and *Notothenia coriiceps* and *Gasterosteus aculeatus*) from timetree^55^ used as the calibration time points, we estimated the divergent time between each species by MCMCtree from the PAML package^56^ with default parameters.

### Synteny analysis

The synteny analysis of *D. undecimradiatus* against *L. crocea* and *T. rubripes* was performed on both whole-genome nucleotide level and gene level. On nucleotide level, we used Lastz (v1.02.00)^57^ to identify synteny blocks with parameters “T = 2 C = 2 H = 2000 Y = 3400 L = 6000 K = 2200”, and aligned blocks with length less than 1 kb were filtered.

On gene level, we used JCVI (v0.8.12)^58^ to identify synteny genes based on CDS. On JCVI pipeline, sequence alignment was carried out using Lastal (v979) with parameters “-u 0 -P 48 -i3G -f BlastTab”, and then the results were filtered by C-score with parameters “C-score > = 0.70 tandem_Nmax=10”. Finally, we filtered out the syntenic gene blocks spanning less than 5 genes.

### Construction of gene trees

To construct a gene tree that truly reflects the evolutionary history, the gene synteny results were used to accurately select orthologous genes. Only genes on 1:1:1 syntenic blocks were extracted and the human gene set was used as an outgroup. For each orthologous gene cluster, we used MUSCLE (v3.8.31)^26^ to do multiple sequence alignments of CDS with default parameters, and then used RAxML (v8.2.12)^54^ to construct ML tree using the GTRCATX model with parameters “-f a -x 12345 -p 12345 -# 100”.

### Population demographic history inference

The history of effective population size was reconstructed using PSMC (v0.6.5-r67)^20^. Firstly, diploid genome reference for the individual was constructed using SAMtools and BCFtools^59^ with parameters “samtools mpileup -C30” and “vcfutils.pl vcf2fq -d 10 -D 100” separately. Then, the demographic history was inferred using PSMC with “−N25 −t15 −r5 −p 4 + 25*2 + 4 + 6” parameters. The estimated generation time (g) and mutation rate per generation per site (μ) were set to 1 and 2.5e^−8^.

### Expansion and contraction of gene families and construction of the phylogenetic tree of gene family

Expansion and contraction of each gene family were identified by Café (v2.1)^60^ based on the time-calibrated tree. To obtain potential functions of the gene families, the number of different KO terms was counted for each gene family. The functions of the gene families were assigned by the corresponding KO terms with the highest count. The KEGG pathways involved by KO terms were extracted for further functional analysis. To construct phylogenetic trees of gene families, the CDS sequences were extracted to construct maximum likelihood (ML) tree using RAxML (v8.2.12)^54^.

## Supplementary information

Supplementary Information.

## Data Availability

The sequencing data and genome sequences of Mekong tiger perch have been deposited in NCBI under BioProject accession PRJNA574247. The datasets reported in this study are also available in the CNGB under accession number CNP0000691
