# Supplementary material for: The genome of Mekong tiger perch (Datnioides undecimradiatus) provides insights into the phylogenetic position of Lobotiformes and biological conservation
Source: Sci Rep. 2020 May 18;10:8164. doi: 10.1038/s41598-020-64398-2 (PMC7235238; doi:10.1038/s41598-020-64398-2)
Supplement: Supplementary file 1 — Supplementary Information. [file 41598_2020_64398_MOESM1_ESM.pdf]

The genome of Mekong tiger perch (*Datnioides undecimradiatus*) provides insights into the phylogenetic position of Lobotiformes and biological conservation

Shuai Sun[1,2,3], Yue Wang[1,2,3], Xiao Du[1,2,3], Lei Li[1,2,3,4], Xiaoning Hong[1,2,3,5], Xiaoyun Huang[1,2,3], He Zhang[1,2,3], Mengqi Zhang[1,2,3], Guangyi Fan[1,2,3], Xin Liu[1,2,3,*] & Shanshan Liu[1,2,3,*]

[1] BGI-Qingdao, BGI-Shenzhen, Qingdao, 266555, China

[2] BGI-Shenzhen, Shenzhen, 518083, China

[3] China National GeneBank, BGI-Shenzhen, Shenzhen, 518120, China

[4] School of Future Technology, University of Chinese Academy of Sciences, Beijing 101408, China

[5] BGI Education Center, University of Chinese Academy of Sciences, Shenzhen, 236009, China

[*] Correspondence authors: E-mails: liuxin@genomics.cn; liushanshan@genomics.cn

Supplementary Figures

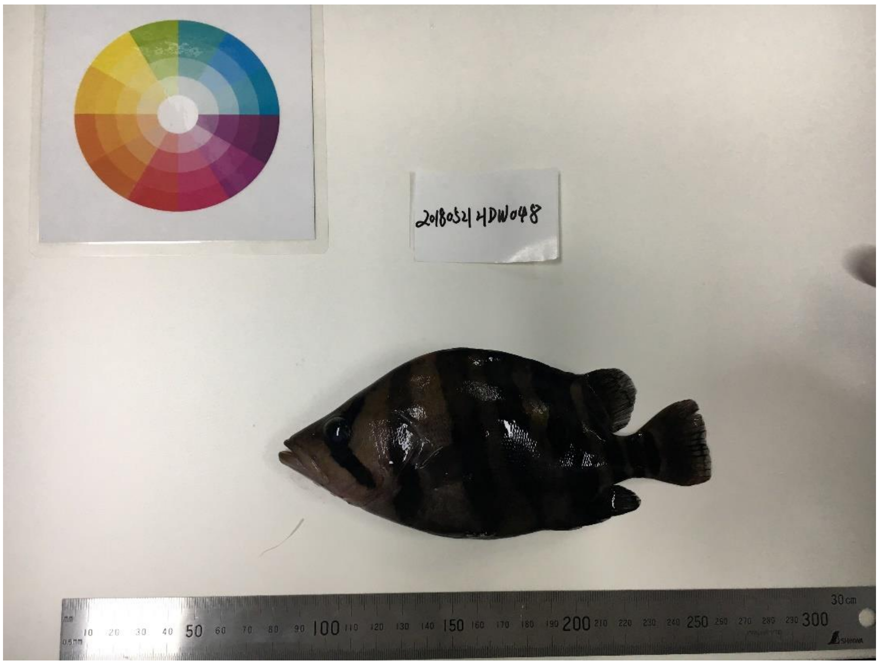

Supplementary Figure S1. The photo of the sequenced Mekong tiger perch captured in Mekong river. The fish had been dead for a while when the samples were obtained. In addition, the picture was taken at night, resulting in pictures that were not very clear. In the figure, light-colored bands are actually white-yellow and dark-colored bands are black.

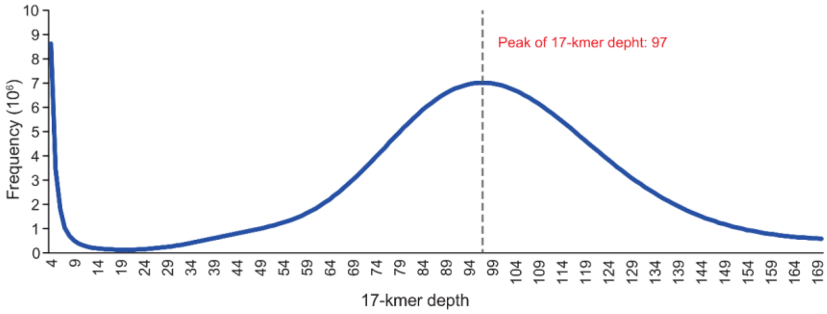

Supplementary Figure S2. k-mer (K=17) frequency at 17-kmer depth. The total number of 17-kmer was 60,437,782,622, and the 17-kmer depth peak was 97. The genome size was estimated by using the formula: genome size = k-mer num/Peak depth. The genome size was estimated to be 623,069,924 bp.

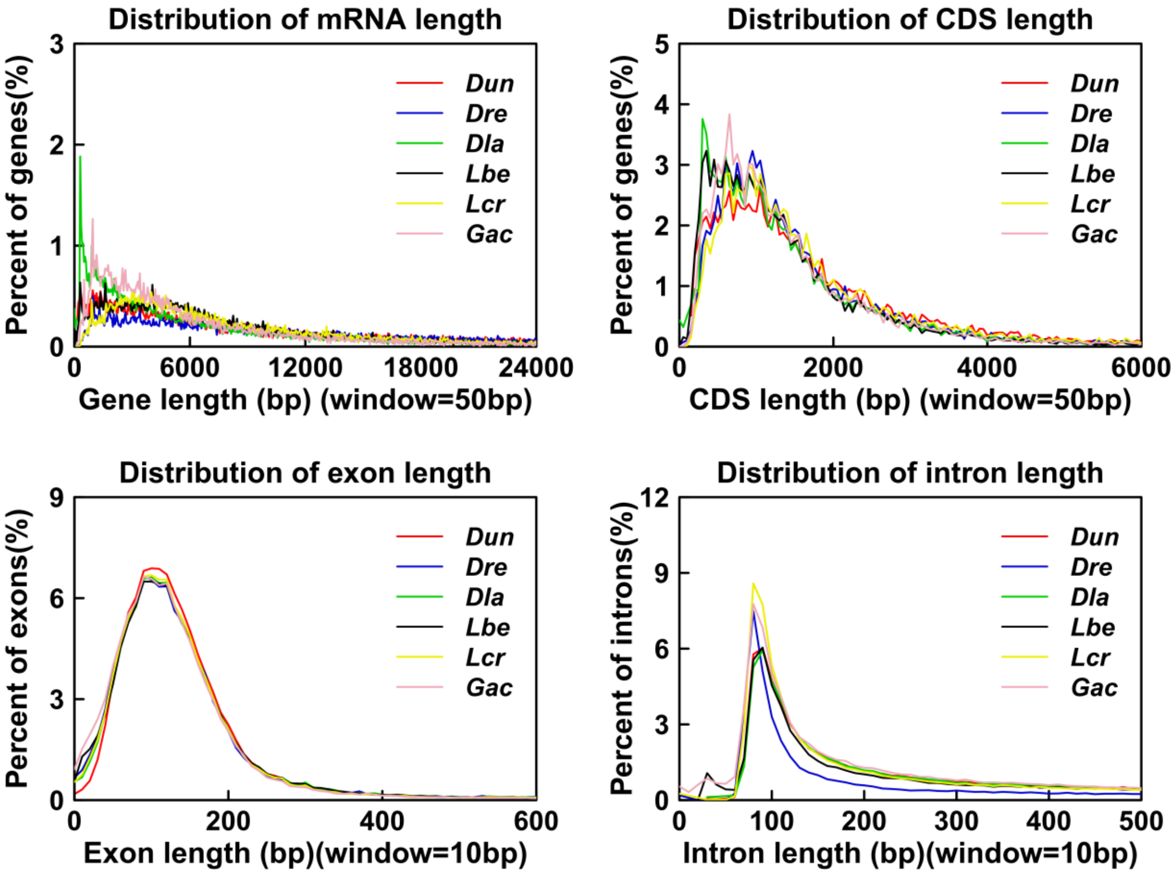

Supplementary Figure S3. The distributions of gene length, CDS length, exon length and intron length of the Mekong tiger perch compared to related species. Scientific names are abbreviated as follows: *Dun*, *Datnioides undecimradiatus*; *Dre*, *Danio rerio*; *Dla*, *Dicentrarchus labrax*; *Lbe*, *Labrus bergylta*; *Lcr*, *Larimichthys crocea*; *Gac*, *Gasterosteus aculeatus.*

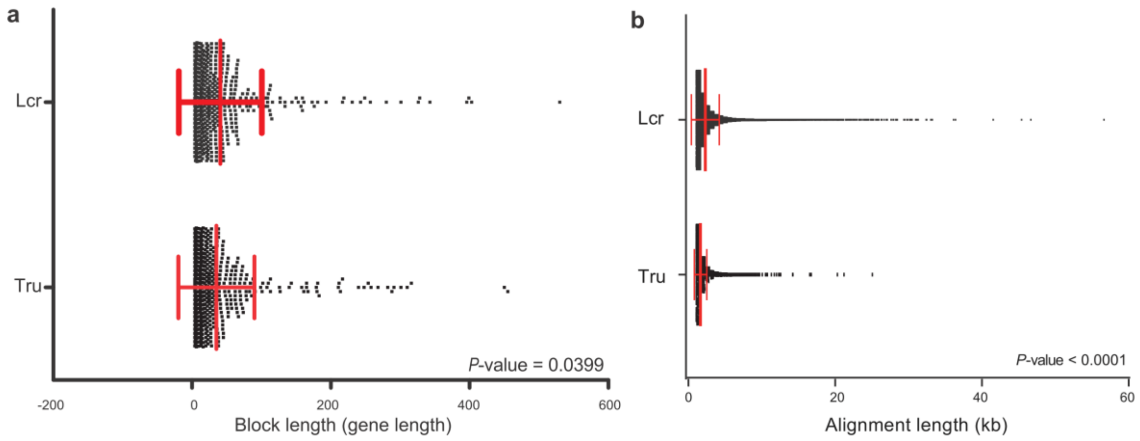

Supplementary Figure S4. The t-test statistics of alignment length and block length. (**a**) The distribution of the length of synteny blocks at gene-level. The individual data points were plotted, and the error bars represent means and standard deviation (SD). *P*-value was calculated using t-statistic. (**b**) The distribution of the length of synteny blocks at nucleotide-level. The individual data points were plotted, and the error bars represent means and standard deviation (SD). *P*-value was calculated using t-statistic.

Supplementary Tables

Supplementary Table S1. The details of the longest 72 scaffolds.

Scaffold ID	Length (bp)	GC content (%)	N ratio (%)
scaffold22	39,224,198	42.35	0.11
scaffold23	24,801,986	42.35	0.46
scaffold24	23,285,271	42.04	0.11
scaffold63	22,554,533	42.44	0.00
scaffold26	19,825,295	41.46	0.03
scaffold27	19,535,368	42.14	0.03
scaffold58	17,458,660	41.69	0.69
scaffold25	17,086,057	41.35	0.04
scaffold28	15,592,228	41.59	0.15
scaffold45	14,958,317	41.94	0.01
scaffold60	13,306,067	42.11	0.00
scaffold61	13,180,949	42.71	0.15
scaffold77	11,668,685	42.18	0.71
scaffold79	11,312,907	42.26	0.03
scaffold59	10,946,362	41.76	1.01
scaffold88	10,679,062	41.98	0.34
scaffold89	9,970,283	43.73	0.36
scaffold62	9,689,676	42.26	0.03
scaffold64	9,424,767	41.08	0.00
scaffold80	9,070,138	41.94	0.00
scaffold90	8,882,121	44.26	1.31
scaffold66	7,972,025	42.51	0.09
scaffold67	7,967,159	42.52	0.09
scaffold155	7,834,019	44.21	0.00
scaffold76	7,191,970	42.34	0.01
scaffold83	7,002,700	43.43	0.00
scaffold86	6,998,795	44.24	0.23
scaffold129	6,961,984	43.28	0.01
scaffold87	6,927,338	42.61	0.02
scaffold85	6,839,013	43.20	0.18
scaffold75	6,655,465	40.74	0.15
scaffold102	6,514,877	42.52	0.00
scaffold78	6,493,655	40.02	0.00
scaffold65	6,204,524	41.27	0.01
scaffold110	6,166,521	42.56	0.05
scaffold81	6,027,087	40.33	0.01
scaffold107	5,522,839	43.61	0.00
scaffold82	5,473,613	45.39	0.01
scaffold108	5,032,825	42.29	0.00

scaffold111	4,849,441	43.54	0.00
scaffold105	4,495,604	42.88	0.01
scaffold101	4,408,335	46.34	0.34
scaffold176	4,273,251	46.55	0.35
scaffold125	4,194,082	46.18	0.00
scaffold84	3,956,156	40.28	0.07
scaffold106	3,626,111	43.70	0.00
scaffold148	3,228,188	41.05	0.00
scaffold147	3,053,217	47.22	0.16
scaffold103	2,975,078	45.96	3.32
scaffold69	2,811,551	45.34	0.10
scaffold68	2,808,140	45.32	0.10
scaffold131	2,720,669	42.90	1.47
scaffold186	2,708,081	39.59	0.00
scaffold200	2,396,806	46.13	0.00
scaffold113	2,371,716	42.63	0.14
scaffold122	2,166,398	46.71	1.88
scaffold201	2,158,157	43.20	0.01
scaffold104	2,153,116	41.93	0.00
scaffold133	2,098,158	41.93	1.91
scaffold18494	1,967,540	45.80	0.15
scaffold18500	1,965,268	45.79	0.15
scaffold124	1,950,648	46.51	0.00
scaffold109	1,680,825	42.16	0.00
scaffold112	1,675,258	41.45	0.00
scaffold137	1,626,245	42.62	2.69
scaffold173	1,556,222	43.15	0.00
scaffold130	1,509,788	41.75	0.00
scaffold185	1,501,851	43.70	0.43
scaffold136	1,478,497	43.30	0.66
scaffold135	1,458,194	47.15	3.18
scaffold174	1,388,139	42.32	0.00
scaffold127	1,380,814	46.71	0.03

Supplementary Table S2. Repeat annotation of the Mekong tiger perch genome.

Type	Repbase TEs		TE proteins		De novo		Combined TEs	
	**Length (bp)**	**% in genome**	**Length (bp)**	**% in genome**	**Length (bp)**	**% in genome**	**Length (bp)**	**% in genome**
DNA	17,221,407	2.89	1,702,129	0.29	29,460,021	4.95	36,076,902	6.06
LINE	7,375,913	1.24	4,399,417	0.74	12,426,966	2.09	16,022,028	2.69
SINE	669,112	0.11	-	0.00	779,372	0.13	1,135,924	0.19
LTR	6,635,295	1.12	2,003,642	0.34	6,211,489	1.04	10,973,341	1.84
Other	5,519	0.00	-	0.00	-	0.00	5,519	0.00
Unknown	0	0.00	-	0.00	11,324,183	1.90	11,324,183	1.90
Total	27,747,330	4.66	8,099,032	1.36	53,245,130	8.95	60,091,293	10.10

Supplementary Table S3. The statistics of predicted genes using different methods.

Method	Software/Species	Number of predicted genes	Average gene length (bp)	Average CDS length (bp)	Average exon number	Average exon length (bp)	Average intron length (bp)
ab initio	Augustus	27,725	11,086	1,405	8.09	173.65	1,364.94
	Genscan	30,673	13,954	1,574	9.08	173.29	1,531.77
Homolog-based	*Danio rerio*	47,021	20,926	2,036	11.87	171.58	1,738.22
	Dicentrarchus labrax	28,410	12,929	1,482	8.17	181.52	1,597.33
	Labrus bergylta	39,430	21,442	1,500	8.72	172.08	2,583.80
	Larimichthys crocea	47,335	19,977	2,193	12.7	172.68	1,520.11
	Gasterosteus aculeatus	29,039	10,617	1,458	8.96	162.8	1,150.80
Combined	EVM	21,160	14,391	1,846	10.88	167.25	1,457.20

Supplementary Table S4. ncRNA annotation of the Mekong tiger perch genome.

**Type**	**Sub-type**	**Copy number**	**Average length (bp)**	**Total length (bp)**	**% of genome**
miRNA	-	262	82.65	21654	0.003639
tRNA	-	800	76.11	60886	0.010232
rRNA	rRNA	145	149.87	21731	0.003652
	18S	12	388.33	4660	0.000783
	28S	47	215.02	10106	0.001698
	5.8S	7	117.14	820	0.000138
	5S	79	77.78	6145	0.001033
snRNA	snRNA	277	128.78	35671	0.005995
	CD-box	117	94.60	11068	0.00186
	HACA-box	77	154.45	11893	0.001999
	splicing	71	141.03	10013	0.001683

Supplementary Table S5. Genes annotated on mitochondrial genome.

Mitochondrial genome	Start	End	Length(bp)	Direction	Type	Gene name	Gene product	Occurred Counts
C515351	134	202	69	+	tRNA	trnF(gaa)	tRNA-Phe	1
C515351	202	1170	969	+	rRNA	s-rRNA	12S ribosomal RNA	1
C515351	1170	1242	73	+	tRNA	trnV(uac)	tRNA-Val	1
C515351	1262	2954	1693	+	rRNA	l-rRNA	16S ribosomal RNA	1
C515351	2954	3028	75	+	tRNA	trnL(uaa)	tRNA-Leu	2
C515351	3028	4003	976	+	CDS	ND1	NADH dehydrogenase subunit 1	1
C515351	4008	4079	72	+	tRNA	trnI(gau)	tRNA-Ile	1
C515351	4078	4149	72	-	tRNA	trnQ(uug)	tRNA-Gln	1
C515351	4148	4220	73	+	tRNA	trnM(cau)	tRNA-Met	1
C515351	4220	5267	1048	+	CDS	ND2	NADH dehydrogenase subunit 2	1
C515351	5266	5338	73	+	tRNA	trnW(uca)	tRNA-Trp	1
C515351	5338	5407	70	-	tRNA	trnA(ugc)	tRNA-Ala	1
C515351	5408	5481	74	-	tRNA	trnN(guu)	tRNA-Asn	1
C515351	5516	5585	70	-	tRNA	trnC(gca)	tRNA-Cys	1
C515351	5585	5655	71	-	tRNA	trnY(gua)	tRNA-Tyr	1
C515351	5656	7207	1552	+	CDS	COX1	cytochrome c oxidase subunit I	1
C515351	7207	7278	72	-	tRNA	trnS(uga)	tRNA-Ser	2
C515351	7281	7354	74	+	tRNA	trnD(guc)	tRNA-Asp	1
C515351	7362	8061	700	+	CDS	COX2	cytochrome c oxidase subunit II	1
C515351	8053	8128	76	+	tRNA	trnK(uuu)	tRNA-Lys	1

C515351	8129	8297	169	+	CDS	ATP8	ATP synthase F0 subunit 8	1
C515351	8314	8971	658	+	CDS	ATP6	ATP synthase F0 subunit 6	1
C515351	8970	9756	787	+	CDS	COX3	cytochrome c oxidase subunit III	1
C515351	9755	9827	73	+	tRNA	trnG(ucc)	tRNA-Gly	1
C515351	9827	10178	352	+	CDS	ND3	NADH dehydrogenase subunit 3	1
C515351	10176	10245	70	+	tRNA	trnR(ucg)	tRNA-Arg	1
C515351	10245	10542	298	+	CDS	ND4L	NADH dehydrogenase 4L	1
C515351	10535	11921	1387	+	CDS	ND4	NADH dehydrogenase 4	1
C515351	11916	11984	69	+	tRNA	trnH(gug)	tRNA-His	1
C515351	11984	12051	68	+	tRNA	trnS(gcu)	tRNA-Ser	2
C515351	12055	12128	74	+	tRNA	trnL(uag)	tRNA-Leu	2
C515351	12128	13967	1840	+	CDS	ND5	NADH dehydrogenase subunit 5	1
C515351	13963	14485	523	-	CDS	ND6	NADH dehydrogenase 6	1
C515351	14486	14555	70	-	tRNA	trnE(uuc)	tRNA-Glu	1
C515351	14560	15721	1162	+	CDS	CYTB	cytochrome b	1
C515351	15701	15771	71	+	tRNA	trnT(ugu)	tRNA-Thr	1
C515351	15770	15840	71	-	tRNA	trnP(ugg)	tRNA-Pro	1

Supplementary Table S6. Relationship among CGI density, GC content, gene density and repeat content.

**Pairs**	**Pearson r**	**Lower 95% CI**	**Upper 95% CI**	***P*-value**
CGI density vs. GC content	0.836	0.824	0.848	0.00E+00
CGI density vs. Gene density	0.100	0.059	0.139	1.34E-06
CGI density vs. Repeat content	0.407	0.372	0.440	3.07E-95
GC content vs. Gene density	0.015	-0.025	0.055	4.72E-01
GC content vs. Repeat content	0.363	0.327	0.397	1.12E-74
Gene density vs. Repeat content	-0.055	-0.095	-0.015	6.87E-03

Supplementary Table S7. Nine species used in our study.

**Three letter code**	**Scientific name**	**Common name**	**Order**	**Data source**	**Accession ID**
Ofa	*Oplegnathus fasciatus*	barred knifejaw	Centrarchiformes	NCBI	GCA_003416845.1
Lbe	*Labrus bergylta*	ballan wrasse	Labriformes	NCBI	GCF_900080235.1
Dun	*Datnioides undecimradiatus*	Mekong tiger perch	Lobotiformes	our study	our study
Dla	*Dicentrarchus labrax*	European seabass	Moronidae	NCBI	GCA_000689215.1
Gac	*Gasterosteus aculeatus*	three-spined stickleback	Perciformes	NCBI	GCA_000180675.1
Nco	*Notothenia coriiceps*	black rockcod	Perciformes	NCBI	GCF_000735185.1
Ssc	*Sebastes schlegelii*	Schlegel's black rockfish	Perciformes	NCBI	GCA_004335315.1
Lcr	*Larimichthys crocea*	large yellow croaker	Sciaenidae	NCBI	GCF_000972845.2
Tru	*Takifugu rubripes*	Torafugu	Tetraodontiformes	NCBI	GCF_000180615.1
Sau	*Sparus aurata*	gilthead sea bream	Spariformes	NCBI	GCF_900880675.1

Supplementary Table S8. The statistics of gene family clusters.

Species	Genes* number	Un-clustered genes number	Families number	Unique family's number	Average genes per family
Gac	20,942	1,189	8,775	22	2.25
Dla	25,020	1,411	10,079	67	2.34
Ssc	24,094	416	9,721	21	2.44
Lbe	27,305	870	10,022	132	2.64
Lcr	23,163	267	9,984	23	2.29
Nco	22,099	2,671	9,761	135	1.99
Tru	20,434	691	9,347	37	2.11
Ofa	21,901	1,988	9,464	73	2.1
Sau	25,995	320	10,056	55	2.55
Dun	19,794	1,707	8,934	100	2.02

* Pseudo-genes (stop-gained in middle) were filtered out, and the genes with protein length <50 were also removed.

Supplementary Table S9. Distribution of synteny of alignment statistics at whole genes level at different cutoffs.

Species aligned to ***Dun***	Cutoff of block length*	Synteny coverage (%)	Block number	Average block length	Median block length	Min block length	Max block length
Lcr	>= 4	96.20	499	40.79	23	4	530
(gene	>= 10	93.33	395	49.99	30	10	530
number:		76.32	198	81.57	52	30	530
23423)	>= 30						
Tru	>= 4	91.60	552	35.11	17	4	455
(gene	>= 10	87.15	400	46.1	25.5	10	455
number:		67.98	169	85.12	56	30	455
28679)	>= 30						

* block length was defined as the number of spanning genes on *D. undecimradiatus*.

Supplementary Table S10. Synteny of alignment statistics at whole-genome nucleotide sequences level.

**Species aligned to *Dun***	**Cutoff of alignment length**	**Synteny coverage (%)**	**Alignment number**	**Average alignment length (bp)**	**Median alignment length (bp)**	**Min alignment length (bp)**	**Max alignment length (bp)**
Lcr (length:658 M)	≥ 1kb	41.13%	105,447	2,321	1,717	1,000	56,540
	≥ 2kb	26.02%	41,422	3,738	2,967	2,000	56,540
	≥ 5kb	8.89%	6,811	7,769	6,571	5,000	56,540
Tru (length:391 M)	≥ 1kb	10.04%	36,032	1,657	1,388	1,000	24,987
	≥ 2kb	3.58%	7,312	2,552	2,954	2,000	24,987
	≥ 5kb	0.39%	352	6,621	5,952	5,000	24,987

Supplementary Table S11. Genome assembly and gene set quality assessed by BUSCO.

		Genome level		Gene level	
		Larimichthys crocea	*Takifugu rubripes*	*Larimichthys crocea*	*Takifugu rubripes*
actinopterygii (db_v9)	Complete BUSCOs (C)	4362 (95.2%)	4263 (93.0%)	4554 (99.3%)	4345 (94.7%)
	Complete and single-copy BUSCOs (S)	4275 (93.3%)	4168 (90.9%)	2615 (57.0%)	3000 (65.4%)
	Complete and duplicated BUSCOs (D)	87 (1.9%)	95 (2.1%)	1939 (42.3%)	1345 (29.3%)
	Fragmented BUSCOs (F)	119 (2.6%)	202 (4.4%)	23 (0.5%)	134 (2.9%)
	Missing BUSCOs (M)	103 (2.2%)	119 (2.6%)	7 (0.2%)	105 (2.4%)
	Total BUSCO groups searched	4584	4584	4584	4584
metazoa (db_v9)	Complete BUSCOs (C)	918 (93.9%)	915 (93.6%)	973 (99.5%)	950 (97.1%)
	Complete and single-copy BUSCOs (S)	882 (90.2%)	880 (90.0%)	656 (67.1%)	670 (68.5%)
	Complete and duplicated BUSCOs (D)	36 (3.7%)	35 (3.6%)	317 (32.4%)	280 (28.6%)
	Fragmented BUSCOs (F)	7 (0.7%)	12 (1.2%)	4 (0.4%)	15 (1.5%)
	Missing BUSCOs (M)	53 (5.4%)	51 (5.2%)	1 (0.1%)	13 (1.4%)
	Total BUSCO groups searched	978	978	978	978

Supplementary Table S12. Genes involved in the pigment development.

**Pigmentary function**	**Gene symbols**	**Genes**
Components of melanosomes	gpnmb	evm.model.scaffold89.24
Components of melanosomes	slc24a4	evm.model.scaffold22.131;
Components of melanosomes	trpm1	evm.model.scaffold27.487;
Components of melanosomes	tspan10	evm.model.scaffold80.229;
Components of melanosomes	vat1	evm.model.scaffold18344.71;evm.model.scaffold18367.69;evm.model.scaffold60.236
Iridophores	chm	evm.model.scaffold200.145
Iridophores	csf1	evm.model.scaffold64.448
Iridophores	ece2	evm.model.scaffold75.239
Iridophores	fbxw4	evm.model.scaffold111.35;
Iridophores	fhl2	evm.model.scaffold61.509;evm.model.scaffold63.296;evm.model.scaffold82.226
Iridophores	foxd3	evm.model.scaffold23.901;
Iridophores	gart	evm.model.scaffold82.268;
Iridophores	Ltk	evm.model.scaffold61.336;
Iridophores	med12	evm.model.scaffold81.240;
Iridophores	mpv17	evm.model.scaffold18251.25;evm.model.scaffold18349.23;
Iridophores	trim33	evm.model.scaffold158.1;
Iridophores, Melanocyte development	ednrb	evm.model.scaffold76.195;evm.model.scaffold62.302;evm.model.scaffold63.409;
Iridophores, Melanocyte development	impdh1	evm.model.scaffold27.353;evm.model.scaffold66.197;evm.model.scaffold67.196;
Iridophores, Melanocyte development	oca2	evm.model.scaffold23.184
Iridophores, Melanocyte development	sox9	evm.model.scaffold80.182;
Iridophores, Melanocyte development, Xanthophore differentiation	sox10	evm.model.scaffold85.272;
Leucophores, Pteridine synthesis	xdh	evm.model.scaffold82.33;
Leucophores, Xanthophore differentiation	gch1	evm.model.scaffold110.30;
Leucophores, Xanthophore differentiation	pax7	evm.model.scaffold24.275;

Leucophores, Xanthophore differentiation	slc2a11	evm.model.scaffold60.596;evm.model.scaffold24.381;evm.model.scaffold24.795;evm.model.scaffold26.142
Melanocyte development	adam17	evm.model.scaffold61.347;
Melanocyte development	adamts20	evm.model.scaffold27.709;
Melanocyte development	adrb2	evm.model.scaffold62.231;
Melanocyte development	apc	evm.model.scaffold155.410;
Melanocyte development	atp6v0b	evm.model.scaffold75.6;
Melanocyte development	bcl2	evm.model.scaffold75.147;
Melanocyte development	brsk2	evm.model.scaffold27.208;evm.model.scaffold89.353
Melanocyte development	c10orf11	evm.model.scaffold28.337;evm.model.scaffold28.342
Melanocyte development	cited1	evm.model.scaffold157.20
Melanocyte development	creb1	evm.model.scaffold63.159;
Melanocyte development	dct	evm.model.scaffold63.418;
Melanocyte development	dock7	evm.model.scaffold23.909;evm.model.scaffold80.316
Melanocyte development	eda	evm.model.scaffold62.157
Melanocyte development	edar	evm.model.scaffold63.630;
Melanocyte development	edn3	evm.model.scaffold24.1046;evm.model.scaffold64.123;
Melanocyte development	ednrb2	evm.model.scaffold173.15;evm.model.scaffold90.41
Melanocyte development	egfr	evm.model.scaffold75.24;
Melanocyte development	en1	evm.model.scaffold83.123
Melanocyte development	erbb3	evm.model.scaffold24.541;
Melanocyte development	fgfr2	evm.model.scaffold28.677;
Melanocyte development	frem2	evm.model.scaffold25.244;evm.model.scaffold58.224;evm.model.scaffold88.161
Melanocyte development	fzd4	evm.model.scaffold58.625;
Melanocyte development	gata3	evm.model.scaffold17418.1;
Melanocyte development	gfpt1	evm.model.scaffold176.182;
Melanocyte development	gja5	evm.model.scaffold63.370;
Melanocyte development	gli3	evm.model.scaffold45.346;
Melanocyte development	gnaq	evm.model.scaffold26.243;
Melanocyte development	gpc3	evm.model.scaffold62.322;evm.model.scaffold62.324;
Melanocyte development	gpr161	evm.model.scaffold63.326;
Melanocyte development	hdac1	evm.model.scaffold87.53;
Melanocyte development	hps1	evm.model.scaffold28.431;
Melanocyte development	hps4	evm.model.scaffold176.213;
Melanocyte development	hps6	evm.model.scaffold28.443;
Melanocyte development	hsd3b1	evm.model.scaffold121.40

Melanocyte development	igsf11	evm.model.scaffold88.256
Melanocyte development	ikbkg	evm.model.scaffold153.7;
Melanocyte development	irf4	evm.model.scaffold23.1068;
Melanocyte development	itgb1	evm.model.scaffold107.36;evm.model.scaffold45.427
Melanocyte development	kcnj13	evm.model.scaffold148.100
Melanocyte development	kit	evm.model.scaffold23.254;
Melanocyte development	kitlg	evm.model.scaffold27.925
Melanocyte development	lmx1a	evm.model.scaffold23.711;evm.model.scaffold80.296
Melanocyte development	mbtps1	evm.model.scaffold105.8;
Melanocyte development	mcoln3	evm.model.scaffold107.276;evm.model.scaffold23.933
Melanocyte development	mef2c	evm.model.scaffold26.63;
Melanocyte development	mib1	evm.model.scaffold75.31;
Melanocyte development	mib2	evm.model.scaffold130.11;
Melanocyte development	mitf	evm.model.scaffold24.917;evm.model.scaffold90.355
Melanocyte development	mreg	evm.model.scaffold63.744
Melanocyte development	myc	evm.model.scaffold45.322;
Melanocyte development	myo5a	evm.model.scaffold89.43;
Melanocyte development	oprm1	evm.model.scaffold28.210;
Melanocyte development	rab27a	evm.model.scaffold78.237;
Melanocyte development	recql4	evm.model.scaffold79.292
Melanocyte development	rnf41	evm.model.scaffold90.252;
Melanocyte development	scarb2	evm.model.scaffold155.132;
Melanocyte development	scg2	evm.model.scaffold147.42;evm.model.scaffold75.225
Melanocyte development	sf3b1	evm.model.scaffold63.23;
Melanocyte development	sfxn1	evm.model.scaffold28.394;evm.model.scaffold62.97
Melanocyte development	skiv2l2	evm.model.scaffold155.121;
Melanocyte development	slc24a5	evm.model.scaffold104.16;
Melanocyte development	slc45a2	evm.model.scaffold155.130;
Melanocyte development	snai2	evm.model.scaffold75.68;
Melanocyte development	sox18	evm.model.scaffold90.125
Melanocyte development	sox2	evm.model.scaffold23.977;
Melanocyte development	tfap2e	evm.model.scaffold79.450;
Melanocyte development	trpm7	evm.model.scaffold89.41;
Melanocyte development	tyr	evm.model.scaffold58.630;
Melanocyte development	tyrp1	evm.model.scaffold108.177;
Melanocyte development	usp13	evm.model.scaffold23.587;
Melanocyte development	vps11	evm.model.scaffold201.85;
Melanocyte development	vps18	evm.model.scaffold22.483

Melanocyte development	zic2	evm.model.scaffold63.207;
Melanocyte development, Melanosome transport	tpcn2	evm.model.scaffold110.180;
Melanogenesis regulation	asip1	evm.model.scaffold24.1152;
Melanogenesis regulation	atrn	evm.model.scaffold61.91;evm.model.scaffold61.93
Melanogenesis regulation	clcn7	evm.model.scaffold85.359;
Melanogenesis regulation	corin	evm.model.scaffold103.174;
Melanogenesis regulation	ctns	evm.model.scaffold23.51;
Melanogenesis regulation	drd2	evm.model.scaffold58.505;
Melanogenesis regulation	mc1r	evm.model.scaffold84.143;
Melanogenesis regulation	mgrn1	evm.model.scaffold80.306;
Melanogenesis regulation	myg1	evm.model.scaffold24.229
Melanogenesis regulation	nf1	evm.model.scaffold148.96;
Melanogenesis regulation	ostm1	evm.model.scaffold22.252
Melanogenesis regulation	pah	evm.model.scaffold66.283;evm.model.scaffold67.285;
Melanogenesis regulation	pomc	evm.model.scaffold18287.1;evm.model.scaffold82.36;
Melanogenesis regulation	shroom2	evm.model.scaffold23.206;evm.model.scaffold28.153;
Melanogenesis regulation	slc7a11	evm.model.scaffold109.18;
Melanogenesis regulation	zeb2	evm.model.scaffold63.1009;evm.model.scaffold82.245;
Melanogenesis regulation, Components of melanosomes	rab32	evm.model.scaffold22.284;
Melanogenesis regulation, Components of melanosomes	rab38	evm.model.scaffold58.626;
Melanosome biogenesis	ankrd27	evm.model.scaffold123.74;evm.model.scaffold17829.1;
Melanosome biogenesis	ap1g1	evm.model.scaffold78.212;
Melanosome biogenesis	ap1m1	evm.model.scaffold107.226;
Melanosome biogenesis	ap3b1	evm.model.scaffold186.47;
Melanosome biogenesis	ap3d1	evm.model.scaffold23.686;
Melanosome biogenesis	bloc1s2	evm.model.scaffold23.292;
Melanosome biogenesis	bloc1s3	evm.model.scaffold58.568;
Melanosome biogenesis	bloc1s4	evm.model.scaffold79.226
Melanosome biogenesis	bloc1s6	evm.model.scaffold104.27;
Melanosome biogenesis	cd63	evm.model.scaffold90.258;
Melanosome biogenesis	dtnbp1	evm.model.scaffold79.219;
Melanosome biogenesis	fig4	evm.model.scaffold22.305
Melanosome biogenesis	gpr143	evm.model.scaffold63.395;

Melanosome biogenesis	hps3	evm.model.scaffold23.989;
Melanosome biogenesis	hps5	evm.model.scaffold27.568;
Melanosome biogenesis	kif13a	evm.model.scaffold22.1353;
Melanosome biogenesis	lyst	evm.model.scaffold28.639
Melanosome biogenesis	mlana	evm.model.scaffold155.272;
Melanosome biogenesis	nsf	evm.model.scaffold125.81;evm.model.scaffold85.138
Melanosome biogenesis	rabggta	evm.model.scaffold23.271;
Melanosome biogenesis	th	evm.model.scaffold27.193;
Melanosome biogenesis	txndc5	evm.model.scaffold45.10;
Melanosome biogenesis	vps33a	evm.model.scaffold176.125;
Melanosome biogenesis, Melanocyte development	bloc1s5	evm.model.scaffold45.11;
Melanosome transport	crh	evm.model.scaffold45.154;
Melanosome transport	dctn1	evm.model.scaffold108.42;evm.model.scaffold61.121
Melanosome transport	dctn2	evm.model.scaffold24.217;
Melanosome transport	ippk	evm.model.scaffold173.23;
Melanosome transport	map2k1	evm.model.scaffold27.524;
Melanosome transport	mlph	evm.model.scaffold63.882
Melanosome transport	myo7a	evm.model.scaffold122.10;
Melanosome transport	rab11a	evm.model.scaffold89.340;
Melanosome transport	rab17	evm.model.scaffold63.881;
Melanosome transport	rab1a	evm.model.scaffold106.125;evm.model.scaffold111.24;evm.model.scaffold122.11;evm.model.scaffold135.99
Melanosome transport	rab3ip	evm.model.scaffold68.83;evm.model.scaffold69.84;
Melanosome transport	rab8a	evm.model.scaffold23.677;
Melanosome transport	ric8b	evm.model.scaffold27.301
Melanosome transport	tmem33	evm.model.scaffold86.109
Melanosome transport, Melanosome biogenesis	pmel	evm.model.scaffold24.612;evm.model.scaffold79.47;
Melanosome transport, Melanosome biogenesis	trappc6a	evm.model.scaffold58.569;
Pigment cell differentiation	cdh2	evm.model.scaffold75.131;
Pigment cell differentiation	lef1	evm.model.scaffold25.192;
Pigment cell differentiation	ovol1	evm.model.scaffold131.150;
Pigment cell differentiation, Melanocyte development	wnt3a	evm.model.scaffold75.50;
Pteridine synthesis	gchfr	evm.model.scaffold61.168
Pteridine synthesis	mycbp2	evm.model.scaffold63.234;
Pteridine synthesis	pcbd1	evm.model.scaffold165.14;
Pteridine synthesis	pcbd2	evm.model.scaffold77.416;

Pteridine synthesis	qdpr	evm.model.scaffold86.97;
Pteridine synthesis	spr	evm.model.scaffold26.365;evm.model.scaffold59.406
Pteridine synthesis, Melanogenesis regulation	gart	evm.model.scaffold82.268;
Xanthophore differentiation	ghr	evm.model.scaffold26.345;evm.model.scaffold59.333;
Xanthophore differentiation	leo1	evm.model.scaffold159.5;
Xanthophore differentiation	pax3	evm.model.scaffold147.48;evm.model.scaffold147.49;evm.model.scaffold15214.1;
Xanthophore differentiation	sox5	evm.model.scaffold65.166;

Supplementary Table S13. KEGG pathways involved by the contracted gene families.

Level 1 KEGG pathway	Level 2 KEGG pathway	Number of gene family	Details of gene family (ID)
Cellular Processes	Cell growth and death	10	2348;23;26;18;2368;1576;415;774;2156;493
Cellular Processes	Cell motility	1	5098
Cellular Processes	Cellular community-eukaryotes	4	4060;894;680;2920
Cellular Processes	Transport and catabolism	11	528;958;2678;1949;960;2065;2061;2348;2920;950;529
Environmental Information Processing	Membrane transport	2	3614;2065
Environmental Information Processing	Signal transduction	13	4381;525;894;2065;528;542;680;2156;529;5098;4490;2061;4507
Environmental Information Processing	Signaling molecules and interaction	13	4060;2061;1967;2348;611;521;525;631;616;894;1965;542;680
Genetic Information Processing	Folding, sorting and degradation	1	561
Genetic Information Processing	Replication and repair	3	2065;493;562
Genetic Information Processing	Transcription	1	493
Genetic Information Processing	Translation	4	482;497;2020;4918
Metabolism	Carbohydrate metabolism	2	2065;2061
Metabolism	Global and overview maps	9	2499;2514;2061;2065;6338;2047;2020;3783;3762
Metabolism	Glycan biosynthesis and metabolism	4	497;6338;955;3762
Metabolism	Lipid metabolism	3	3783;2514;2047
Metabolism	Metabolism of cofactors and vitamins	1	2499
Organismal Systems	Aging	1	2514
Organismal Systems	Circulatory system	2	5098;2156
Organismal Systems	Development	2	482;1949

Organismal Systems	Digestive system	7	1021;5098;2156;680;955;894;2065
Organismal Systems	Endocrine system	8	3614;4381;2156;680;2514;4490;5098;2061
Organismal Systems	Environmental adaptation	3	4490;2156;680
Organismal Systems	Immune system	23	493;1965;542;23;26;18;2301;1967;2348;4490;525;1949;4857;528;774;680;4060;529;4855;2368;12264;2565;482
Organismal Systems	Nervous system	6	2514;2061;4490;680;3783;2156
Organismal Systems	Sensory system	7	2621;2514;2617;2061;2619;680;2156

Supplementary Table S14. KEGG pathways involved by the expanded gene families.

Level 1 KEGG pathway	Level 2 KEGG pathway	Number of gene family	Details of gene family (ID)
Cellular Processes	Cell growth and death	2	6680;2948
Cellular Processes	Transport and catabolism	1	2967
Environmental Information Processing	Signal transduction	3	3683;2654;6680
Environmental Information Processing	Signaling molecules and interaction	2	2654;6680
Genetic Information Processing	Folding, sorting and degradation	1	794
Metabolism	Global and overview maps	2	9457;3404
Metabolism	Glycan biosynthesis and metabolism	2	9457;3404
Organismal Systems	Development	2	1171;6680
Organismal Systems	Digestive system	1	3933
Organismal Systems	Endocrine system	1	3683
Organismal Systems	Environmental adaptation	1	2654
Organismal Systems	Immune system	1	6680
Organismal Systems	Nervous system	1	2654
Organismal Systems	Sensory system	1	546

Supplementary Table S15. Functions for the contracted gene families.

Family ID	KO Assigned to gene family	Function of gene family
4	-	-
112	-	-
121	-	-
127	K09228	KRAB, KRAB domain-containing zinc finger protein
139	K09228	KRAB, KRAB domain-containing zinc finger protein
148	K09228	KRAB, KRAB domain-containing zinc finger protein
149	K09228	KRAB, KRAB domain-containing zinc finger protein
276	-	-
400	K04299	P2RY14, purinergic receptor P2Y, G protein-coupled, 14
414	K05051	TAAR, trace amine associated receptor
439	K05051	TAAR, trace amine associated receptor
506	K20865	NLRP12, NACHT, LRR and PYD domains-containing protein 12
508	K22614	NLRC3, NOD3, NLR family CARD domain-containing protein 3
511	K22614	NLRC3, NOD3, NLR family CARD domain-containing protein 3
512	K22614	NLRC3, NOD3, NLR family CARD domain-containing protein 3
537	K01446	PGRP, peptidoglycan recognition protein
539	K06712	BTN, CD277, butyrophilin
556	K06751	MHC1, major histocompatibility complex, class I
557	K06751	MHC1, major histocompatibility complex, class I
603	K06499	CEACAM, CD66, carcinoembryonic antigen-related cell adhesion molecule
608	K06467	CD22, SIGLEC2, CD22 antigen
610	K06467	CD22, SIGLEC2, CD22 antigen
647	-	-
654	K12012	TRIM35, tripartite motif-containing protein 35
663	K12006	TRIM16, tripartite motif-containing protein 16
669	K12006	TRIM16, tripartite motif-containing protein 16
675	K12015	TRIM39, tripartite motif-containing protein 39 [EC:2.3.2.27]
813	K17388	ROCK2, Rho-associated protein kinase 2 [EC:2.7.11.1]
814	-	-
826	-	-
827	K17854	CYP2K, cytochrome P450 family 2 subfamily K
939	-	-
951	K08826	HIPK, homeodomain interacting protein kinase [EC:2.7.11.1]

991	K10380	ANK, ankyrin
1057	K07375	TUBB, tubulin beta
1059	-	-
1100	K07377	NRXN, neurexin
1392	-	-
1492	K03654	recQ, ATP-dependent DNA helicase RecQ [EC:3.6.4.12]
1498	K06560	MRC, CD206, CD280, mannose receptor, C type
1502	-	-
1505	K06560	MRC, CD206, CD280, mannose receptor, C type
1518	K11275	H1_5, histone H1/5
1519	K11252	H2B, histone H2B
1521	K11253	H3, histone H3
1522	K11251	H2A, histone H2A
1533	K18626	TCHH, trichohyalin
1554	K05096	FLT1, VEGFR1, FMS-like tyrosine kinase 1 [EC:2.7.10.1]
1567	K06634	CCNH, cyclin H
1592	K16826	SIRPB2, signal-regulatory protein beta 2
1593	K16826	SIRPB2, signal-regulatory protein beta 2
1597	K10784	TRAV, T cell receptor alpha chain V region
1599	K06553	VPREB, CD179a, pre-B lymphocyte gene
1600	K06856	IGH, immunoglobulin heavy chain
1601	K06856	IGH, immunoglobulin heavy chain
1611	K06554	IGLL1, IGLL, CD179b, immunoglobulin lambda-like polypeptide 1
1613	K06553	VPREB, CD179a, pre-B lymphocyte gene
1614	K10785	TRBV, T-cell receptor beta chain V region
1634	K09228	KRAB, KRAB domain-containing zinc finger protein
1659	K04257	OLFR, olfactory receptor
1664	K04257	OLFR, olfactory receptor
1666	K04257	OLFR, olfactory receptor
1769	K19470	C1QTNF6, complement C1q tumor necrosis factor-related protein 6
1807	K06238	COL6A, collagen, type VI, alpha
2057	-	-
2080	-	-
2117	K01068	ACOT1_2_4, acyl-coenzyme A thioesterase 1/2/4 [EC:3.1.2.2]
2142	K04615	GABBR, gamma-aminobutyric acid type B receptor
2146	K22611	SART3, TIP110, squamous cell carcinoma antigen recognized by

		T-cells 3
2159	K16810	TBCCD1, TBCC domain-containing protein 1
2200	K18543	HCE, choriolysin H [EC:3.4.24.67]
2614	K14480	APOL, apolipoprotein L
2787	K06719	CD300, CD300 antigen
3054	K06556	CD200, CD200 antigen
3071	K06733	SLAMF7, CD319, SLAM family member 7
3082	-	-
3084	-	-
3480	K05039	SLC6A6S, solute carrier family 6 (neurotransmitter transporter, GABA) member 6/8/11/12/13
3550	-	-
3561	-	-
3620	-	-
3651	K18684	ALOXE3, hydroperoxy icosatetraenoate dehydratase/isomerase [EC:4.2.1.152 5.4.4.7]
3796	K05152	TNFRSF14, HVEM, CD270, tumor necrosis factor receptor superfamily member 14
3883	-	-
3919	K06087	CLDN, claudin
4277	-	-
4321	K13826	HBZ, hemoglobin subunit zeta
4384	-	-
4401	K17072	IFI47, interferon gamma inducible protein 47
4503	K21922	KCTD21, BTB/POZ domain-containing protein KCTD21
4741	K04612	CASR, calcium-sensing receptor
4743	K04612	CASR, calcium-sensing receptor
4827	K11170	DHRSX, dehydrogenase/reductase SDR family member X [EC:1.1.-.-]
4846	K06554	IGLL1, IGLL, CD179b, immunoglobulin lambda-like polypeptide 1
6363	K03663	FUT9, 4-galactosyl-N-acetylglucosaminide 3-alpha-L-fucosyltransferase [EC:2.4.1.152]
7663	K14217	IFIT1, interferon-induced protein with tetratricopeptide repeats 1
11586	-	-
11850	-	-
11970	K14639	SLC15A5, solute carrier family 15, member 5
12008	K09228	KRAB, KRAB domain-containing zinc finger protein

12055	K03985	PLAUR, CD87, plasminogen activator, urokinase receptor

Supplementary Table S16. Functions for the expanded gene families.

Family ID	KO Assigned to gene family	Function of gene family
382	K10594	HERC1, E3 ubiquitin-protein ligase HERC1 [EC:2.3.2.26]
686	K10651	TRIM21, SSA1, tripartite motif-containing protein 21 [EC:2.3.2.27]
1838	K22614	NLRC3, NOD3, NLR family CARD domain-containing protein 3
2386	K04275	LPAR4, GPR23, lysophosphatidic acid receptor 4
2388	-	-
2448	K08826	HIPK, homeodomain interacting protein kinase [EC:2.7.11.1]
2918	K04202	MC4R, melanocortin 4 receptor
3120	K06840	SEMA3, semaphorin 3
5371	-	-
6325	-	-
6345	K06065	NCOR2, SMRT, nuclear receptor co-repressor 2
6410	-	-
7362	-	-
7948	K05131	IFNAR2, interferon receptor 2
8246	-	-
8505	K02366	EXT1, glucuronyl/N-acetylglucosaminyl transferase EXT1 [EC:2.4.1.224 2.4.1.225]
8556	-	-
8643	-	-
12055	-	-
